# Treatment related toxicities with combination BRAF and MEK inhibitor therapy in resected stage III melanoma

**DOI:** 10.3389/fonc.2022.855794

**Published:** 2022-09-23

**Authors:** Morgan Homan, Govind Warrier, Christopher D. Lao, Sarah Yentz, Shawna Kraft, Leslie A. Fecher

**Affiliations:** ^1^ Department of Pharmacy, University of Michigan, Ann Arbor, MI, United States; ^2^ Department of Internal Medicine, University of Michigan, Ann Arbor, MI, United States; ^3^ Department of Internal Medicine and Dermatology, University of Michigan, Ann Arbor, MI, United States

**Keywords:** BRAF, MEK, melanoma, pyrexia, toxicity, inhibitor

## Abstract

Combination dabrafenib (D) and trametinib (T) is an FDA approved adjuvant therapy for patients with resected stage III BRAF-mutant melanoma. We describe treatment-related toxicities with adjuvant D+T in a real-world population through a retrospective case series. The primary endpoint was development of toxicities.

**Results:**

Eighteen of the 20 patients (90%) required at least one treatment interruption due to adverse events (AEs), 11 patients (55%) required a dose reduction and 13 (65%) permanently discontinued therapy due to an AE. The nine patients who did not require dose reduction had been initiated on a lower starting dose of dabrafenib. The most common treatment-limiting AEs were recurrent pyrexia and chills (85%) and liver laboratory abnormalities (50%). The median total time on therapy was 148.5 days (range 19-383), 40.7% (range 5.2-100%) of the intended one-year duration.

**Conclusion:**

Adjuvant treatment of melanoma with combination D+T is associated with treatment-limiting toxicities in the majority of this patient group. Patients should be carefully monitored throughout therapy.

## Introduction

Invasive melanoma represents approximately 1% of all skin cancers but accounts for the majority of skin cancer related deaths. For localized melanoma, surgical resection alone has been the standard of care with high 5-year melanoma specific survival rates for early stages. Patients with stage II and III disease are at higher risk for recurrence after resection with some cases progressing to metastatic melanoma. 5-year survival rates for melanoma metastatic regionally and distantly are 66% and 27%, respectively (1).

In melanoma, mutations in BRAF are found in approximately 40% of cases and result in constitutive activation of the MAPK pathway (2). Mutant BRAF, and downstream kinase protein MEK, have proved viable targets for melanoma therapies. Three combinations of inhibitors of mutant BRAF and MEK have been approved by the FDA for treatment of advanced unresectable melanoma (3).

A study of adjuvant combination therapy with BRAF inhibitor, dabrafenib, and MEK inhibitor, trametinib, in patients with resected stage III BRAF V600E/K-mutant melanoma showed improved recurrence free survival benefit at 3 years with overall survival rate of 86% compared to 77% with placebo. This study was published in September 2017 and led to FDA approval in April 2018. The difference in 3 year overall survival was not considered statistically significant as it did not cross the prespecified interim analysis boundary of P=0.000019 for significance (4, 5). In the aforementioned study, adverse events led to dose interruption in 66% of patients, dose reduction in 38% of patients, and permanent discontinuation of therapy in 26% of patients. Common adverse events include fevers and chills, with any grade reported in 63% of patients. These data are greater than that reported in the metastatic setting (3). Subsequent analyses suggest the rate of adverse events decreased with increased duration of therapy (6, 7). Adjuvant therapy with dabrafenib and trametinib has an intended duration of one year (4, 5).

Here we present a retrospective case series of patients with resected stage III melanoma treated with adjuvant BRAF and MEK inhibition with the purpose of describing toxicities in a real-world population.

## Methods

We reviewed medical records of patients treated at a single center with resected stage III melanoma who started treatment with adjuvant dabrafenib and trametinib by three independent academic medical oncologists between November 2017 and December 2019. Planned treatment was for a total of 1 year of dabrafenib and trametinib with full doses being dabrafenib 150 mg twice daily and trametinib 2 mg daily. Primary endpoint was development of toxicities. Using REDCap Data Management software, baseline patient characteristics were collected in addition to secondary endpoints including number of treatment interruptions, dose reductions, and total time on combination therapy. The study was performed according to a protocol approved by the institutional review board at the University of Michigan.

## Results

### Patient characteristics

Twenty patients were treated with adjuvant dabrafenib and trametinib during the study period (Supplementary Appendix 1). Stage at the time of initiation of adjuvant therapy is reported; in the event of relapse, this incorporates pathology from both the initial presentation and at relapse.

### Individual patient treatment courses

**Table 1** reviews the adjuvant treatment course for each patient. Eighteen patients (90%) required at least one treatment interruption due to adverse events. Fifteen patients required two or more interruptions (mean = 2). The median time to first interruption was 30.5 days (range 3-94 days). Eleven patients required a dose reduction, with median time to first reduction of 40 days (range 8-138 days). The nine patients who did not require a dose reduction had all been initiated on a lower dose of dabrafenib (75 mg BID) due to physician experience with toxicities in prior patients. Fourteen patients were started at a reduced dabrafenib dose of 75 mg twice daily. All patients except two patients were started on full dose of trametinib (2 mg daily). Patients were evaluated for uptitration of doses throughout their yearlong course. While no specific criteria for uptitration were used, if patients had good tolerance of current doses without recent need for holding or dose reduction, an increase in dose was considered. Five patients were uptitrated, two to the full dose of dabrafenib (150mg twice daily). Side effects prevented uptitration in the remaining nine patients. Additionally, two of the patients who were started at a lower dose of dabrafenib also required dose reduction of trametinib (0.5 mg once daily for one patient and 2 mg every other day for another patient). All patients have either completed or discontinued therapy.

**Table 1:** Treatment summaries.

Pt	Age	Sex	Stage, AJCC 8^th^ ed.	Starting Dabrafenib (D) Dose	Starting Trametinib (T) Dose	Max tolerated dose (D,T)^a^	Total time (days) on combination therapy	Percentage of time on intended therapy^b^	Time (days) to first hold	Time (days) to first dose reduction	Reason for discontinuation	Relapse after adjuvant theraphy	Days to Relapse^h^	Current Status; treatment
1	54	F	IIIC	150 mg BID	2 mg daily	150 mg BID, 2 mg daily	85	23.2%	27	32	pyrexia, chills, elevated liver labs	No	N/A	NED/Obs
2	40	M	IIIC	150 mg BID	2 mg daily	75 mg BID, 2 mg daily	40	11%	3	8	pyrexia, chills, elevated liver labs	unknown	N/A	Lost to follow up
3	40	F	IIIC	150 mg BID	2 mg daily	75 mg BID, 2 mg daily	57	15.6%	13	15	pyrexia, chills, elevated liver labs	No	N/A	Ned/Obs
4	73	F	IIIC	75 mg BID	2 mg daily	75 mg BID, 2 mg daily	67	18.4%	49	N/A	pyrexia, chills, demand ischemia^c^	Yes -In scar and LN	550	Additional relapse, Systemic treatment
5	62	F	IIIB	75 mg BID	2 mg daily	75 mg BID, 2 mg daily	333	91.2%	49	62	Recurrent URI requiring holds- attributed to tobacco/pulmonary status	Yes -lung	427	Systemic therapy
6	50	F	IIIB	75 mg BID	2 mg daily	150 mg qam 75 mg qpm, 2 mg daily	184	50.4%	18	22	decrease in RV function^d^	No	N/A	NED/Obs
7	64	M	IIIC	75 mg BID	2 mg daily	75 mg BID, 2 mg daily	38	10.4%	9	N/A	Poor tolerance, patient preference, dilated right atrium^e^	No	N/A	NED/Obs
8	68	F	IIIB	150 mg BID	2 mg daily	Not reached	19	5.2%	5	10	pyrexia, chills, elevated liver labs, thrombocytopenia, arthralgias	No	N/A	NED/Obs
9	58	M	IIIC	150 mg BID	2 mg daily	150 mg qam 75 mg qhs, 2 mg daily	365	100%	55	67	Completed course	Yes brain	28	DOD
10	46	M	IIIC	75 mg BID	2 mg daily	150 mg qam 75 mg qhs, 2 mg daily	366	100%	29	40	Completed course	Yes -lung and LNs	142	Systemic therapy
11	32	F	IIIA	75 mg BID	2 mg daily	75 mg BID, 2 mg daily	64	17.5%	37	N/A	pyrexia, diarrhea, abdominal pain	Yes -LN	444	NED; s/p systemic therapy and surgery
12	29	M	IIIC	75 mg BID	2 mg daily	150 mg qam 75 mg qhs, 2 mg daily	373	100%	62	N/A	Compled course	No	N/A	NED/Obs
13^f^	43	M	IIIC	75 mg BID	2 mg QOD	75 mg BID, 2 mg QOD	50	13.7%	26	49	Pyrexia, chills	No	N/A	NED/Obs
14	21	F	IIIC	75 mg BID	2 mg QOD	75 mg BID, 2 mg QOD	113	31%	25	N/A	Pyrexia, chills	No	N/A	NED/Obs
15	47	M	IIIB	75 mg BID	2 mg daily	75 mg BID, 2 mg daily	32	8.8%	32	N/A	RA and RV dilation dizziness, nausea, fatigue	No	N/A	NED/Obs
16^f^	62	M	IIID	75 mg BID	2 mg daily	150 mg BID, 2 mg daily	195	53.4%	N/A	N/A	Disease progression	Disease progression	N/A	s/p Systemic therapy/ CR
17	34	IIIC	M	75 mg BID	2 mg daily	150 mg BID, 2 mg daily	377	100%	N/A	N/A	Completed course; simultaneous relapse	Yes -local	N/A	Systemic treatment for additional relapse
18	38	F	IIIB	150 mg BID	2 mg daily	150 mg BID, 2 mg daily	363	99.4%	94	115	Completed course	No	N/A	NED/Obs
19	40	M	IIIC	75 mg BID	2 mg daily	75 mg BID, 2 mg daily	352^g^	96.4%	38	N/A	Pyrexia, chills, elevated liver labs	Yes -brain	1167	Surgery, radiation, systemic therapy
20	40	M	IIIA	75 mg BID	2 mg QOD	75 mg BID, 2 mg QOD	383	100%	60	138	Completed course	No	N/A	NED/Obs

Patients 3,4,7,9,17,18,19, and 20 did not have CLND. N/A, Not Applicable.

a Max tolerated dose: Stable on dose for 14 days without adverse events.

b Intended therapy: one year of combination therapy at any doses; Percentage of time on intended therapy: number of days on combination therapy out of 365 days 100% is maximum.

c Demand ischemia secondary to pyrexia and chills.

d RV function normalized on repeat imaging off medication.

e Initial determination of right atrium dilation was found to be unchanged from baseline after review by a second provider.

BID, twice daily; NED, no evidence of disease; Obs., close observation; D + T, dabrafenib and trametinib; RA, right atria; RV, right ventricle.

f Received adjuvant radiation.

g includes 45 day hold required for elevated liver enzymes.

h Days to relapse = time relapse first occurred from day of last dose of adjuvant therapy.

### Adverse events

The adverse events experienced by our patients are compared to phase III trial toxicity data in **Table 2**. Recurrent pyrexia and chills occurred in 17 patients (85%) and was the primary reason for treatment discontinuation in nine patients (45%). Ten patients (50%) experienced liver laboratory elevations, with median maximum values for the first reporting were AST of 81 (range: 44-550 IU/L), ALT of 95 (range: 27-470 IU/L), and alkaline phosphatase of 150 (range: 102-544 IU/L). This contributed to discontinuation in five patients (25%).

**Table 2:** Select adverse events.

	Long et al. (4)		Study population		
	N = 435		N = 20		
	Patients, n (%)				
**Adverse Event**	**Total (Any Grade)**	**Grade 3-4**	**Total (Any Grade)**	**Grade 1-2**	**Grade 3-4**
Liver laboratory Abnormalities	ALT: 67 (15%) AST: 63 (14%)	16 (4%)	10 (50%)	8 (40%)	2 (10%)
Pyrexia	273 (63%)	23 (5%)	17 (85%)	16 (80%)	1 (5%)^a^
Chills/Rigors	161 (37%)	6 (1%)	17 (85%)	17 (85%)	0
Nausea	172 (40%)	4 (1%)	12 (60%)	12 (60%)	0
Arthralgias	120 (28%)	4 (1%)	9 (45%)	9 (45%)	0
Rash	106 (24%)	0	9 (45%)^b^	9 (45%)	0
Vomiting	122 (28%)	4 (1%)	4 (20%)	4 (20%)	0
Cardiac abnormalities	Not reported	Not reported	4 (20%)	4 (20%)	0
Vision complaint	Not reported	Not reported	1 (5%)^c^	1 (5%)	0

Grading with CTCAE v5.0 where applicable

a Grade 3 pyrexia = >40C

b Including panniculitis vs. erythema nodosum and maculopapular rash

c Patient described vision change as an inability to see the end of a word during the process of reading because the word “looked bright”. Similar concerns were not subsequently described.

## Discussion

Our study offers a novel examination of a real-world population with resected stage III melanoma treated with adjuvant combination BRAF and MEK inhibition. In our experience, adjuvant combination BRAF and MEK inhibition was associated with clinically significant treatment related toxicities with the rate of adverse events exceeding what has been reported in the literature. Specifically, in the COMBI-AD trial, fevers and chills of all grades occurred in 63% of patients. Dose interruption in 66% of patients, dose reduction in 38% of patients, and permanent discontinuation of therapy in 26% of patients (4). The median time to onset of pyrexia was 23 days with median duration of 3 days. Of the patients who experienced pyrexia, 72% had recurrence (≥ 2 episodes) (8). Interestingly, there was no difference in patient reported quality of life between those receiving treatment and those receiving placebos.

In our population, 90% of patients experienced adverse effects prompting treatment interruption, 55% required at least one dose reduction, and 65% permanently discontinued therapy due to an adverse event. For the 20 patients who completed or discontinued therapy, the median total time on therapy was 148.5 days, 40.7% of the intended duration. The majority of these patients never tolerated the FDA labeled combination doses.

Our systematic approach to BRAF/MEK therapy includes obtaining baseline labs including complete blood count (CBC), comprehensive metabolic panel (CMP), lactate dehydrogenase (LDH), EKG for QTc assessment, echocardiogram to evaluate cardiac function, ophthalmologic evaluation, and standard cross-sectional imaging to assess disease at baseline. Cardiovascular testing (EKG and echocardiogram) is repeated every 3-6 months or with change in clinical status given potential cardiovascular adverse events (9). History and physical exams with laboratory testing are repeated every 4 weeks, however, additional 2 week appointments have been necessary due to symptoms and lab abnormalities. Specific attention is given to changes in liver labs which have been seen in several patients despite the relative infrequency reported in advanced melanoma patients. It is possible that use of acetaminophen could have contributed to these liver laboratory abnormalities. Combination therapy was initially started at doses of dabrafenib 150 mg BID and trametinib 2 mg daily. High rates of poor tolerance often related to pyrexia and chills leading to need for treatment interruptions, re-evaluations within days and weeks of starting treatment, some emergency department evaluations, as well as multiple dose reductions, and early termination of treatment were frequently observed regardless of disease and/or patient characteristics, or treating physician. This physician group discussed these issues and elected to decrease the starting dose of dabrafenib to 75 mg BID with the intent of minimizing initial treatment related adverse events, minimizing treatment interruptions, with the intent of uptitrating to the goal dose of 150 mg BID. This generally allowed improved adherence and fewer toxicities although uptitration was not tolerated in most cases.

Recommended management of common side effects with combination BRAF and MEK inhibition has been described in metastatic melanoma and are being used in the adjuvant setting as well (10–12). As in clinical trials, dose interruptions and dose reductions of one or both medications, as well as supportive medications, were used in the management of adverse events. In this patient cohort, premedication for pyrexia/chills with acetaminophen, ibuprofen, or low dose prednisone (n=2) were typically used for recurrent episodes of pyrexia/chills. The majority of patients used both acetaminophen and NSAIDs. This permitted some patients to tolerate the desired dose of therapy. In the two patients who received prednisone 5mg daily, this did not permit tolerance and both discontinued treatment prematurely. To date, eight of the 20 patients have relapsed with only one death. Two of these relapsed on therapy. The impact of dose interruptions and/or dose reductions on outcomes remains to be determined in the adjuvant setting. Data in advanced melanoma have shown inferior outcomes with intermittent dosing of BRAF and MEK combination therapy (13, 14).

There are few other reports of patient groups treated with adjuvant dabrafenib and trametinib. One report of 36 patients had incidences of fevers or chills of all grades of 36% and 2.8%, respectively (15). This group also reported liver adverse events in 11% as well as SAEs in 22.2% and a ‘protocol completion rate’ of 64.3%. There was no comment on cardiac side effects. Another report of 65 patients reported a discontinuation rate due to treatment related adverse events of only 9% and only one patient stopped treatment due to pyrexia (16). Forty-three patients completed treatment as scheduled. The median time to discontinuation was 9 months. The incidence of chills was very low at 1.5% and of fever at 35.4%. There was no specific comment on liver or cardiac toxicities, nor on dosages or dose reductions or interruptions. It is not known as to the reason for the differences seen in the few papers published on real world experience with dabrafenib/trametinib for melanoma in the adjuvant setting however it is possible that there is a geographical factor. Dabrafenib can cause hemolytic anemia in patients who have G6PD deficiency and it is known that there are geographic differences worldwide in percent of patients who are G6PD deficient (17, 18). While no patients at our institution experienced hemolytic anemia, this is one example of possible geographic differences in medication tolerance.

Oncologists continuously face the difficult task of balancing benefits and toxicities associated with cancer treatment. We report our findings of the side effects of adjuvant combination BRAF and MEK inhibition to demonstrate the frequency and severity of toxicities. Adjuvant combination BRAF and MEK inhibition is an approved treatment for resected stage III melanoma but requires diligent toxicity assessment and management.

## Limitations

There were some limitations to our study. First, this study was performed at a single academic center. Second, this study was retrospective and information bias must be considered. Third, practice pattern variation among physicians in dosing, management of toxicities, and decisions to hold therapy or dose reduce is probable.

## Conclusions

Our findings indicate that adjuvant combination dabrafenib and trametinib in the treatment of resected stage III melanoma can be associated with treatment limiting toxicities. We support a comprehensive approach to adjuvant treatment including a thorough initial evaluation, close monitoring for toxicities, and prompt interventions with the goal of completing therapy with tolerable adverse events. Additional studies with larger numbers of patients are needed to validate our findings.

## Data availability statement

The original contributions presented in the study are included in the article/**Supplementary Material**. Further inquiries can be directed to the corresponding author.

## Ethics statement

The studies involving human participants were reviewed and approved by University of Michigan IRB. Written informed consent for participation was not required for this study in accordance with the national legislation and the institutional requirements.

## Author contributions

MH, GW, SK, and LF contributed to conception and design and manuscript drafting. MH, GW, SK and LAF performed data collection. All authors provided substantial contribution to data analysis, editing, and final approval of the manuscript.

## Conflict of interest

LF: institutional funding for clinical trials (site PI) from Array/Pfizer, Bristol Myers Squibb, EMD Serono, Kartos. Consultant for Elsevier. Array/Pfizer funding of ECOG-ACRIN clinical trial (national PI) for LAF.

The remaining authors declare that the research was conducted in the absence of any commercial or financial relationships that could be construed as a potential conflict of interest.

## Publisher’s note

All claims expressed in this article are solely those of the authors and do not necessarily represent those of their affiliated organizations, or those of the publisher, the editors and the reviewers. Any product that may be evaluated in this article, or claim that may be made by its manufacturer, is not guaranteed or endorsed by the publisher.
