# Supplementary material for: Treatment related toxicities with combination BRAF and MEK inhibitor therapy in resected stage III melanoma
Source: Front Oncol. 2022 Sep 23;12:855794. doi: 10.3389/fonc.2022.855794 (PMC9538392; doi:10.3389/fonc.2022.855794)
Supplement: Supplementary file 1 [file Table_1.pdf]

Supplementary Appendix 1. Patient Characteristics

Characteristic	Number of patients (%)
Median age, years (range)	44.9 (21-73)
Sex	
Male	11 (55%)
Female	9 (45%)
ECOG Performance Status	
0	18 (90%)
1	2 (10%)
$\geq$2	0
Stage (AJCC 8th Edition)	
IIIA	2 (10%)
IIIB	5 (25%)
IIIC	12 (60%)
IIID	1 (5%)
BRAF	
V600E	18 (90%)
V600K	2 (10%)
Positive Lymph Nodes	
1	9 (45%)
2 or 3	7 (35%)
4+	4 (20%)
Primary Tumor Ulceration	6 (30%)
Satellite or In-Transit Metastases	5 (25%)
Complete Lymph Node Dissection	12 (60%)
Prior Adjuvant Systemic Therapy	1 (5%)[a]
Radiation Therapy	2 (10%)

Underlying Liver Disease	2 (10%)[b]
Baseline Acetaminophen Use	13 (65%)
Baseline Statin Therapy	2 (10%)
Alcohol Use	
Yes - Less than 2 drinks per day	12 (60%)
Yes - 2 or more drinks per day	2 (10%)
No	5 (25%)
Not reported	1 (5%)

[a]Received adjuvant interferon with prior melanoma occurrence
[b]Hepatic steatosis
